# The psychological resources of success: satisfaction with academic majors, psychological capital, and achievement motivation among future tourism and hospitality leaders

**DOI:** 10.3389/fpsyg.2024.1501861

**Published:** 2025-01-03

**Authors:** Abdulaziz Mohammed Alismail, Mazen Omar Almulla, Abdullah Saleh Mohammed Albohnayh, Ahmed Hassan Abdou

**Affiliations:** ^1^Department of Education and Psychology, College of Education, King Faisal University, Al-Ahsa, Saudi Arabia; ^2^Department of Social Studies, College of Arts, King Faisal University, Al-Ahsa, Saudi Arabia

**Keywords:** PsyCap, motivation to achieve, major satisfaction, tourism and hospitality education, Saudi Arabia

## Abstract

**Introduction:**

Exploring the factors that drive academic achievement motivation is a vital area in educational psychology, particularly within specialized fields like tourism and hospitality higher education. Achievement motivation, essential for academic and career success, is shaped by various positive psychological resources and contextual factors. Grounded in the framework of positive psychology, this study examines how satisfaction with academic majors (SAT) predicts achievement motivation among tourism and hospitality students in Saudi Arabia. Additionally, the research investigates the mediating role of psychological capital (PsyCap) - a positive psychological construct encompassing hope, optimism, resilience, and self-efficacy - within this relationship.

**Methods:**

A quantitative approach was employed using Partial Least Squares Structural Equation Modeling (PLS-SEM). Data were collected from 522 junior and senior undergraduate students enrolled in tourism and hospitality programs across Saudi Arabia. Validated scales were used to measure SAT, PsyCap, and achievement motivation.

**Results:**

The findings indicate that greater satisfaction with one’s academic major (SAT) positively predicts both PsyCap and achievement motivation. Furthermore, PsyCap was found to be positively and significantly associated with achievement motivation and partially mediate the relationship between SAT and achievement motivation.

**Conclusion:**

This study’s findings underscore the vital role of positive psychological resources in empowering students and advancing motivation, offering a validated model that informs future educational studies and interventions within tourism and hospitality while underscoring the significance of fostering psychological well-being in higher education.

## 1 Introduction

The tourism and hospitality sector has emerged as a dynamic and competitive field, requiring specialized knowledge and considerable psychological resilience and motivation in its future leaders (Chung-Herrera et al., 2003; Ho and Law, 2017; Sharma and Munjal, 2023). As academic institutions strive to prepare students for successful careers in this sector, understanding the psychological factors that contribute to fostering their motivation to achieve educational and professional goals becomes increasingly essential. One such area of interest is the interplay between students’ satisfaction with their chosen academic majors (SAT), psychological capital (PsyCap), and their achievement motivation—a critical determinant of academic success and future career aspirations. Exploring the interplay between these variables is vital because it reveals specific mechanisms through which satisfaction with academic majors influences achievement motivation, informing targeted interventions.

While earlier research has established the significance of SAT and PsyCap independently, integrating satisfaction with academic majors, PsyCap, and achievement motivation is not widely covered in existing research, particularly in tourism and hospitality education, to capture the complexity of these interactions. More specifically, the gap in this research lies in the limited understanding of the specific mechanisms by which satisfaction with academic majors influences achievement motivation. Although the individual impacts of academic satisfaction and psychological capital on achievement motivation have been studied, the intermediating effect of PsyCap in this nexus remains underexplored. Understanding how PsyCap mediates this relationship can provide deeper insights into the psychological resources that motivate tourism and hospitality students.

Hence, the current study aims to explore the complex interplay between students’ satisfaction with their academic majors (SAT), psychological capital (PsyCap), and achievement motivation. Specifically, it examines the direct impacts of academic major satisfaction on both students’ PsyCap and their achievement motivation. Additionally, the study intends to investigate the role of PsyCap in influencing achievement motivation and analyze its mediating effect on the relationship between SAT and achievement motivation. To achieve the previous objectives, we seek to answer the following questions. What are the direct effects of academic major satisfaction on students’ psychological capital and achievement motivation? What role does psychological capital play in fostering motivation among students who are satisfied with their chosen field of study? How does PsyCap mediate the nexus between SAT and achievement motivation?

In this study, we will focus on tourism and hospitality majors in Saudi Arabia. The focus on tourism and hospitality in this study is primarily motivated by the critical role these sectors play in Saudi Arabia’s economic diversification efforts as outlined in the Saudi Vision 2030. Tourism and hospitality education in Saudi Arabia is a rapidly evolving field driven by the country’s vision to diversify its economy and decrease oil dependency (Abdullah Al-Malki, 2023). The Saudi Vision 2030 initiative has significantly emphasized developing the tourism and hospitality sector, recognizing its potential to create jobs, attract international visitors, and promote cultural exchange (Pavan, 2023). As part of Saudi Vision 2030, the tourism industry is vital for generating jobs. The government intends to boost employment in the field by nearly 50% to reach 1.5 million jobs by 2020 (Shabir and Sharma, 2019). This strategic focus has led to substantial investments in educational programs and institutions aimed at preparing a skilled workforce for the growing industry.

This study contributes to the literature by addressing a gap in the current research concerning the integration of SAT, PsyCap, and achievement motivation, particularly in the context of tourism and hospitality education. The results of this research will enrich the academic field by highlighting the significant role of SAT in fostering students’ PsyCap and achievement motivation and exploring the substantial effect of PsyCap as a mediator in the academic major satisfaction-achievement motivation relationship. By examining the mediating role of PsyCap, the study offers valuable insights into the psychological factors that drive student motivation and success, thereby enriching the existing body of knowledge in educational psychology and tourism and hospitality education. From a practical point of view, this study will also offer practical implications for tourism and hospitality educators and policymakers in designing programs and policies that cultivate an encouraging and motivating academic environment for future leaders in tourism and hospitality. This study is unique in its focus on the specific interactions between SAT, PsyCap, and achievement motivation, particularly within the relatively under-researched field of tourism and hospitality education. Unlike previous studies that have examined these constructs in isolation (i.e., Baek et al., 2007; Ritonga et al., 2023; Elom et al., 2023; Chi and Lee, 2022; Nambudiri et al., 2020; Khajavy et al., 2019), this research integrates them to reveal the complex mechanisms that influence student outcomes.

## 2 Theoretical background and hypotheses development

### 2.1 Satisfaction with academic majors (SAT) and achievement motivation

SAT is crucial to the broader student experience in higher education, playing a significant role in fostering positive educational outcomes and well-being. Grounded in positive psychology, Nauta (2007) described it as the extent to which students find contentment with their chosen fields of study; it reflects the alignment and fulfillment individuals derive from their academic pursuits. Various studies have pointed to its close relationship with positive outcomes such as academic performance, achievement motivation, subjective well-being, engagement, retention, and overall life satisfaction (Kim et al., 2019; Kim and Lee, 2015; Guo and Wu, 2023; Abuaddous et al., 2021; Kim and Jung, 2023; Madison et al., 2018; Franzen et al., 2021; Mihanović et al., 2016; Milsom and Coughlin, 2015). When students feel satisfied with their selected area of study, they are tended to be motivated, engaged, and committed to achieving their academic and professional goals, aligning with positive psychology’s goal of fostering life satisfaction and personal fulfillment. However, the mechanisms through which major satisfaction influences achievement motivation are not fully understood.

Student achievement motivation, a core construct in positive psychology, is a complex psychological concept that reflects an individual’s drive, desire, and persistence to excel in academic pursuits (Smith et al., 2021; Dwijuliani et al., 2021). It encompasses the internal and external determinants that lead individuals to set and strive for academic goals, engage in learning activities, and persist in facing challenges (Krou et al., 2021; Ahmad et al., 2022). Within a positive education framework, achievement motivation is not merely about striving for grades; it’s about fostering resilience, persistence, and a sense of purpose in educational endeavors, which aligns with positive psychology’s objectives of developing intrinsic motivation and personal strengths (Singh, 2011; Bakar et al., 2010; Bipp and van Dam, 2014). Therefore, exploring the factors influencing achievement motivation, such as satisfaction with academic majors (SAT), becomes essential for educational institutions that aim to cultivate not only skilled but also motivated and well-rounded.

Studies by Nauta (2007), Kim and Lee (2015), and Heo (2016) emphasized the connection between satisfaction with majors and academic achievement and performance, highlighting the foundational role satisfaction plays in the educational journey. Satisfaction with academic majors catalyzes intrinsic motivation, the internal drive that propels students toward academic excellence. Students who express higher levels of satisfaction with their majors tend to demonstrate consistently stronger academic performance. This includes higher grades, deeper engagement in coursework, and more remarkable persistence in the face of challenges (Kim et al., 2019; Kim and Lee, 2015). In addition, numerous scholars underscore student satisfaction’s substantial and positive impact on achievement motivation. The evidence connects contentment in academic pursuits and heightened motivation. As students experience satisfaction in their educational journey, their intrinsic drive and enthusiasm for learning are noticeably elevated (Subandi and Hamid, 2021; Suswanto et al., 2017; Ritonga et al., 2023). In the PBL-based clinical practice education context, Baek et al. (2007) demonstrated a significant correlation between SAT and heightened levels of achievement motivation. When students feel validated and aligned in their academic pursuits, they are more inclined to set higher personal goals and work diligently toward their achievement. Therefore, SAT can be viewed not merely as a product of contentment but as a crucial factor that shapes their academic identity and provides a consistent source of motivation throughout their educational journey compared to their counterparts. Consequently, it could be assumed that.

*H1*: Satisfaction with academic majors is significantly associated with students’ achievement motivation.

### 2.2 Satisfaction with academic majors (SAT) and students’ PsyCap

Psychological capital, often called PsyCap, is a favorable psychological condition distinguished by high levels of hope, self-efficacy, resilience, and optimism (Luthans et al., 2004, 2007). These components collectively enhance students’ ability to set and achieve goals, maintain a positive outlook, and recover from setbacks, aligning with the positive psychology framework that emphasizes developing personal strengths and well-being. In the context of higher education, PsyCap significantly predicts various positive academic and personal outcomes, including achievement motivation, which underscores its essential role in fostering a positive educational experience (Wenjiao and Wanbing, 2021; Datu et al., 2018; Adil et al., 2020). In recent years, scholars have delved into the multifaceted dimensions of PsyCap within higher education context, uncovering positive correlations between psychological capital and various aspects of student outcomes, highlighting how this construct supports students’ personal growth and resilience (Datu and Valdez, 2016; Kaur and Kaur, 2023; Lin, 2020; Siu et al., 2014; You, 2016).

The four facets of PsyCap serve as potent personal resources for university students, providing internal strengths that enable them to flourish in academic settings, navigate demands, sustain motivation, and achieve success, in line with positive psychology’s focus on enhancing individual potential (Sweetman and Luthans, 2010; Siu et al., 2014). Earlier research also illustrated that fostering PsyCap throughout the educational ecosystem significantly enhances study engagement, motivation, intrinsic motivation, and learning empowerment, which are all essential for student well-being and intrinsic motivation (Kaur and Kaur, 2023; Siu et al., 2014), self-regulation, and intelligence beliefs (Sheikhi and Shahmorady, 2016), learning empowerment (You, 2016), and student well-being (Nafees and Jahan, 2017). These findings underscore the value of PsyCap as a driver of positive educational outcomes that align with the positive psychology goals of developing well-rounded and resilient learners.

The connection between satisfaction with academic majors and students’ psychological capital is an essential focus in educational psychology and higher education research. Earlier studies have shown that academic major satisfaction, which refers to the extent to which students feel content and aligned with their chosen field of study, is crucial in shaping psychological capital. When students feel satisfaction with their chosen majors, they tend to experience higher levels of psychological capital. This satisfaction arises from aligning their academic pursuits with their interests, talents, and career goals, fostering a sense of purpose, motivation, and engagement—critical elements of psychological capital (Chi and Lee, 2022; Elom et al., 2023). On the other hand, dissatisfaction with academic majors can lead to reduced motivation, increased stress, and poorer academic performance, ultimately weakening students’ psychological capital. Furthermore, data gathered from 726 senior business students indicated that greater SAT significantly enhanced their academic PsyCap, emphasizing how positive emotional experiences within academic contexts can cultivate psychological strengths essential for academic success (Elom et al., 2023). Additionally, the broaden-and-build theory of positive emotions developed by Fredrickson (2001) supports this relationship. The theory posits that positive emotions broaden individuals’ thought-action repertoires, encouraging exploration and engagement with their environment. Over time, these broadened behaviors help individuals build surviving psychological resources. In the context of this study, the positive emotional state generated from academic satisfaction broadens students’ perspectives, fostering proactive problem-solving and resilience—key components of PsyCap that students can rely on when faced with academic challenges.

On the other hand, the same theory may be applied to address the potential for reverse causality between PsyCap and satisfaction with academic majors. More specifically, the theory suggests that the positive emotions associated with high PsyCap (like optimism and hope) may allow students to expand their thought processes and seek meaning in their academic pursuits. This broadened outlook may help them identify areas in their academic field that align with their personal goals, increasing satisfaction with their chosen major. Moreover, conservation of resources theory (COR) also supports this idea by suggesting that students with more psychological resources (such as PsyCap resources) can better cope with academic demands. This ability reduces stress and increases enjoyment, leading to a more satisfying educational experience (Hobfoll, 2010). These findings reinforce the bidirectional relationship between SAT and PsyCap, confirming their robust relationship. However, this study will focus on the impact of academic major satisfaction on PsyCap. Accordingly, we hypothesized that:

*H2*: Academic major satisfaction is significantly associated with students’ PsyCap.

### 2.3 Students’ PsyCap and achievement motivation

Previous studies have shown that PsyCap and achievement motivation are closely related concepts in student performance and well-being. PsyCap, which comprises self-efficacy, optimism, hope, and resilience, is essential in influencing students’ motivation to achieve their academic goals. Within the framework of positive psychology, PsyCap is viewed as a critical resource that empowers students to thrive academically and personally. Studies consistently demonstrated that students with higher PsyCap tend to exhibit stronger achievement motivation (Wenjiao and Wanbing, 2021; Datu et al., 2018; Adil et al., 2020). Students with psychologically solid capital are more apt to establish challenging objectives, persist in difficulties, and maintain a positive outlook toward their academic pursuits which aligns with positive education’s emphasis on fostering strengths-based development and resilience (Nambudiri et al., 2020; Khajavy et al., 2019; Saadat et al., 2019; Mahfud et al., 2024). This is because the components of PsyCap collectively enhance students’ self-confidence, goal-setting and achievement capacities, optimistic outlook, and ability to recover from setbacks. Such attributes are not only fundamental for academic success but also pivotal for cultivating positive emotional states, optimal engagement, school belongingness, and lifelong well-being, as highlighted in the positive psychology literature (Datu and Valdez, 2019; King et al., 2020; Datu et al., 2018). As revealed in the studies by Datu et al. (2018) and Steinmayr et al. (2019), the findings suggest that students with high self-efficacy strongly believe in their capacity to succeed in tasks. This enhances their motivation to engage in and persist with academic activities, leading to improved achievement motivation and academic performance.

Further, hopeful students are goal-oriented and possess the willpower and pathways to achieve their academic objectives. This goal-directed energy and planning significantly enhances their achievement motivation, driving them to pursue and attain their academic goals (Wenjiao and Wanbing, 2021). In line with positive psychology, hope serves as a psychological resource that not only propels students toward their aspirations but also strengthens their ability to overcome challenges in the academic setting. In addition, optimistic students maintain a positive outlook on their academic future, which fuels their motivation to strive for success (King and Caleon, 2021). This positive expectation helps them stay motivated even when faced with challenges, thereby enhancing their achievement motivation (Raza et al., 2021). Lastly, resilience, a key focus in positive psychology, is critical in fostering adaptability and perseverance, enabling students to stay committed to their goals even in the face of setbacks. Earlier research revealed that resilient students are better equipped to handle academic stress and recover from failures, maintaining their motivation to achieve (Saman and Wirawan, 2021; Herdem, 2019). Based on the prior findings, we hypothesized that:

*H3*: Psychological capital is significantly associated with students’ achievement motivation.

### 2.4 The intermediary effect of PsyCap in the nexus between satisfaction with academic majors (SAT) and students’ achievement motivation

In the current study, psychological capital (PsyCap), encompassing self-efficacy, hope, optimism, and resilience, was utilized as a robust theoretical framework for understanding the interplay between satisfaction with academic majors (SAT) and achievement motivation. The findings of the exciting review revealed that SAT in higher education significantly correlated to higher levels of PsyCap (Chi and Lee, 2022; Elom et al., 2023). Further, results revealed that PsyCap has been established as a critical psychological resource that positively influences educational performance, engagement, and motivation (Luthans et al., 2007; Datu et al., 2018; Adil et al., 2020; King et al., 2020; King et al., 2020). Accordingly, we posit that PsyCap may act as a mediator by translating the positive emotional and psychological outcomes of SAT into higher levels of achievement motivation. Satisfied students are more likely to engage actively in their studies, set ambitious goals, and remain persistent in the face of academic challenges, leveraging their PsyCap, which may boost their achievement motivation. Thus, we hypothesized that:

*H4*: PsyCap plays a significant mediating role in the nexus between students’ SAT and their achievement motivation.

**Figure 1:**
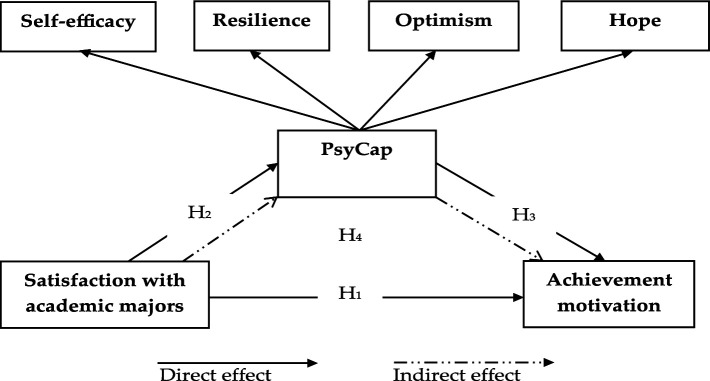
Conceptual framework.

Figure 1 shows the conceptual model used in this study.

## 3 Methodology

### 3.1 Sampling and data collection

The intended participants for this research were hospitality and tourism students, specifically juniors (third year), and seniors (fourth year) aged 18 or above in Saudi Arabia. Participants were chosen using a nonprobability method, specifically the convenience sampling technique. Convenience sampling is often used because it allows for generalizability within uniform groups, such as students focused on a particular area of study (Jager et al., 2017). This study primarily focused on junior and senior students for various reasons. Compared to freshmen (first year) and sophomores (second year), juniors (third year) and seniors (fourth year) have a greater understanding of their major and curriculum. They have completed a substantial portion of their coursework and have more experience with the faculty, peers, and the educational environment. This makes them better suited to provide informed feedback on their satisfaction with their major. Further, junior and senior students are closer to graduation and will likely have a clearer perspective on their academic and career goals. Their motivation to achieve can be more focused, and they may better understand how their major has prepared them for the future.

While the use of convenience sampling may limit the generalizability of study findings, as it relies on a specific group of accessible participants, which may not fully represent the broader population of hospitality and tourism students, the following steps were taken to mitigate this issue. First, we aimed to enhance the representativeness of our sample by including a diverse group of students from multiple academic levels and backgrounds, specifically juniors and seniors, who have substantial experience within their major. Second, we implemented rigorous data collection methods, ensuring high response rates through faculty reminders and clearly informing participants of the study’s purpose to promote honest and thoughtful responses.

Selecting an adequate sample size is crucial to support the validity and reliability of the statistical analysis in this study. Accordingly, following the recommendations of Hair et al. (2019), a minimum sample size of 155 respondents is recommended. This threshold is applicable when the expected path coefficients (*P_min_*) range from 0.11 to 0.20, with a significance level set at 0.05. Additionally, Boomsma (1982) recommends using at least 200 participants for studies employing structural equation modeling to ensure reliable results.

To recruit participants for the survey, faculty members from the tourism and hospitality department at KFU were contacted and requested to allow the recruitment of students for the study. An introductory letter and a Google Forms survey link were provided to participants after they confirmed their willingness to participate. The students received information regarding the study’s objectives, the use of their data, and their freedom to discontinue participation whenever they wished. Further, ethical guidelines were followed during the data collection process including obtaining informed consent from the students, ensuring confidentiality of their responses, and allowing voluntary participation. Faculty members received two email reminders to encourage their students’ participation. From March to May 2024, 522 questionnaires were received and only 501 forms (representing 96%) were found valid for analysis.

### 3.2 The measurements of the study

To fulfill the aim of the study, an online questionnaire was developed and utilized to gather data. The questionnaire form consisted of four sections. The first section listed participants’ socio-demographic information, including gender, academic level, working experience, and future career plan. Sections 2, 3, and 4 demonstrated how the investigated participants perceived SAT, PsyCap, and achievement motivation.

Satisfaction with academic majors (SAT): This measure refers to a student’s positive feelings and contentment regarding their chosen field of study. To assess these feelings, a three-item scale modified by Hong et al. (2023) was applied to measure SAT. A sample of these items is “I am overall satisfied with my academic life in tourism and hospitality management.”

Psychological capital (PsyCap): This measure refers to a positive psychological state characterized by self-efficacy, optimism, hope, and resilience. The school PsyCap scale developed by King and Caleon (2021) was adapted and employed to measure this construct. The scale comprises four dimensions representing PsyCap’s resources. Each dimension includes four items. For example, one self-efficacy item is “I feel confident that I can learn what is taught at university.” A hope item is “If I have problems in university, I can think of many ways to solve them.” An optimism item is “I am optimistic about my future in university,” and a resilience item is, “I do not let study stress affect me too much.”

Achievement motivations (Motv): This measure refers to the intrinsic drive to pursue and attain goals, particularly in academic contexts. A 5-item scale adopted by Chang et al. (2022) was slightly modified and utilized. One of these items includes “No matter how big the obstacles are, I will strive to overcome them when conducting my study.”

Generally, each study item was rated on a 5-point Likert scale, 1 being strongly disagree and 5 being strongly agree. Greater levels of SAT, PsyCap, and achievement motivation among students are reflected in higher average scores.

### 3.3 Data analysis

Descriptive statistics were calculated using SPSS to outline participants’ demographic characteristics and their perceptions of the study constructs. Hypotheses were tested using Partial Least Squares Structural Equation Modeling (PLS-SEM), supported by a bootstrapping approach. PLS-SEM is a statistical technique ideal for exploring complex relationships between observed and latent variables, particularly in exploratory research. We use PLS-SEM for different reasons. It manages models with multiple variables effectively, analyzing direct, indirect, and total effects simultaneously (Hair et al., 2019). This study treated psychological capital (PsyCap) as a higher-order construct, reflecting its multidimensional nature. Further, PLS-SEM does not assume normal data distribution, making it advantageous for datasets with non-normal characteristics (Usakli and Rasoolimanesh, 2023; Dash and Paul, 2021). Finally, PLS-SEM aims to maximize explained variance in dependent variables, enhancing predictive accuracy, which aligns with the study’s objective of understanding factors influencing students’ achievement motivation.

## 4 Results

### 4.1 Participants’ profile

Table 1 presents a demographic analysis of the investigated respondents. Out of the 522 valid responses, over two-thirds were female (*n* = 358, 68.6%), while 31.4% were male. Regarding the academic level, most participants (*n* = 388, 74.3%) were seniors (fourth year), with the remaining 25.7% being juniors (third year). Concerning work experience, 47.7% of respondents reported holding a part-time position in the tourism and hospitality sector, 15.1% held a full-time job, and 37.2% were unemployed. As for their career plans, 75.9% of the respondents intended to pursue a career in the tourism and hospitality sector, and 17% expressed a desire to enroll in a postgraduate program in tourism and hospitality.

**Table 1:** Demographic analysis of the investigated participants.

Characteristic	No.	%
Gender		
Male	164	31.4
Female	358	68.6
Academic level		
Junior	134	25.7
Senior	388	74.3
Working experience		
Full time	79	15.1
Part-time	249	47.7
Un-employed	194	37.2
Career plan		
Seeking a career in the T&H industry	396	75.9
Seeking a career in a field other than T&H	37	7.1
Enrollment in a postgraduate degree in T&H	89	17
Enrollment in a postgraduate degree other than T&H	0	0
Other		
Total	522	100%

### 4.2 Common method variance

Since data gathering was adopted via an online survey, we recognized the potential for common method variance (CMV). To eliminate this, participants’ responses were kept confidential and anonymous, strictly for research objectives (Nancarrow et al., 2001; Randall and Fernandes, 1991). Respondents were also encouraged to answer honestly, a known method for reducing response bias (Phillips and Clancy, 2002). To assess CMV, we performed Harman’s single-factor test, revealing that a single factor explained only 38.7% of the variance—well below the 50% threshold, which suggests CMV was not a significant problem in this research (Podsakoff et al., 2003).

### 4.3 Measurement model assessment

Following the phase of data collection, the psychometric characteristics of the surveyed items were examined, including assessments of reliability, convergent validity, and discriminant validity, utilizing the PLS-SEM algorithm. The findings, as shown in Table 2, indicated favorable psychometric properties. The reliability of the study’s constructs was initially assessed using Cronbach’s alpha to ensure internal consistency, followed by composite reliability (CR) to further confirm reliability (Hair et al., 2019; Sarstedt et al., 2019). As detailed in Table 2, all latent constructs showed Cronbach’s alpha values ranged from 0.860 to 0.963, with CR values from 0.916 to 0.967, exceeding the 0.70 thresholds advised by Hair et al. (2019) and highlighting excellent consistency among the constructs.

**Table 2:** Constructs’ means, standard deviation, reliability, and validity measures.

Construct	Items	M (S.D.)	SFL	Cronbach’s alpha	CR	AVE
Satisfaction with academic major (SAT)	Sat1	4.49 (0.823)	0.809***	0.860	0.916	0.784
	Sat2	4.43 (0.899)	0.939***			
	Sat3	4.26 (1.094)	0.903***			
Psychological capital (PsyCap)				0.963	0.967	0.649
Self-efficacy	PsyCap1	4.42 (0.774)	0.880***	0.902	0.932	0.774
	PsyCap2	4.39 (0.783)	0.898***			
	PsyCap3	4.40 (0.761)	0.806***			
	PsyCap4	4.35 (0.766)	0.930***			
Hope	PsyCap5	4.36 (0.790)	0.850***	0.878	0.919	0.744
	PsyCap6	4.42 (0.799)	0.920***			
	PsyCap7	4.39 (0.799)	0.957***			
	PsyCap8	4.06 (1.067)	0.702***			
Resilience	PsyCap9	4.23 (0.864)	0.957***	0.972	0.979	0.922
	PsyCap10	4.30 (0.858)	0.956***			
	PsyCap11	4.25 (0.848)	0.982***			
	PsyCap12	4.23 (0.811)	0.945***			
Optimism	PsyCap13	4.21 (0.819)	0.957***	0.940	0.957	0.848
	PsyCap14	4.28 (0.822)	0.919***			
	PsyCap15	4.31 (0.842)	0.926***			
	PsyCap16	4.35 (0.836)	0.879***			
Achievement motivation (Motv)	Motv1	4.45 (0.796)	0.884***	0.950	0.962	0.834
	Motv2	4.40 (0.842)	0.946***			
	Motv3	4.48 (0.787)	0.912***			
	Motv4	4.41 (0.780)	0.914***			
	Motv5	4.32 (0.769)	0.909***			

M, mean; S.D., Standard deviation; SFL, standardized factor loading; CR, Composite Reliability; AVE, Average Variance Extracted; ***: *p* < 0.001.

In this study, the constructs’ validity was assessed using both convergent and discriminant measures. Hair et al. (2019) recommended that for convergent validity to be established, standardized factor loading (SFL) should be no less than 0.70, and the AVE must exceed 0.50. Our analysis showed that all study items demonstrated significant standardized factor loadings (SFL) above 0.70, with *p*-values less than 0.001, and the AVE for all latent constructs were between 0.649 and 0.834, significantly higher than the 0.50 criterion, indicating convergent validity has been achieved.

Two methods were applied to verify discriminant validity: the HTMT ratio and the Fornell-Larcker criterion. The Fornell-Larcker guideline states that for adequate discriminant validity, the square root of the AVE of a construct should be higher than its correlations with all other constructs (Fornell and Larcker, 1981). As indicated in Table 3, this criterion was satisfied for all constructs. Furthermore, the HTMT ratios were examined following the 0.85 threshold suggested by Henseler et al. (2015), as depicted in Table 4. All observed HTMT ratios were below this limit, strengthening the evidence for discriminant validity among the constructs in this research.

**Table 3:** Fornell-Larcker discriminant validity.

Construct	Motv	PsyCap	Sat
Achievement motivation (Motv)	**0.913**		
Psychological capital (PsyCap)	0.509^a***^	**0.806**	
Satisfaction with academic majors (Sat)	0.281^a***^	0.339^a***^	**0.885**

The AVE’s square root is depicted by the bolded diagonal values, ^a***^ correlation between latent constructs (***: *p* < 0.001).

**Table 4:** Validity of discrimination via HTMT.

Construct	Motv	PsyCap	Sat
Achievement motivation (Motv)			
Psychological capital (PsyCap)	0.533		
Satisfaction with academic majors (Sat)	0.310	0.373	

An HTMT of 0.85 or less is recommended.

### 4.4 Structural model and hypotheses testing

**Figure 2:**
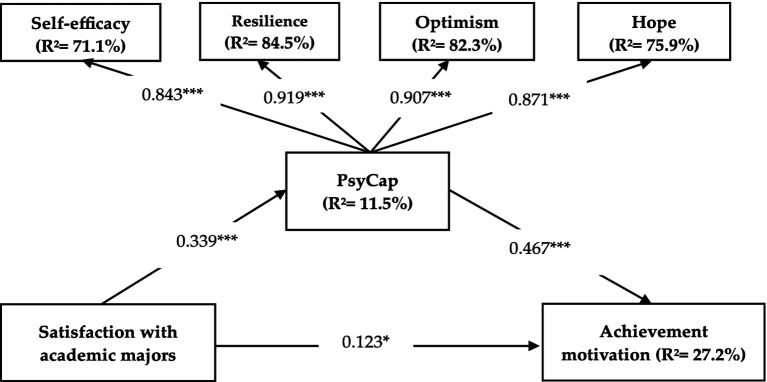
The structural model. Values between paths represent standardized path coefficients, *** = *p* < 0.001, * = *p* < 0.05.

PLS-SEM was employed to examine the research hypotheses. A bootstrapping procedure with 5000 iterations was performed to ensure the robustness of the path coefficient estimates, which indicate the strength and direction of the relationships among constructs. The key findings are summarized in Table 5 and depicted in Figure 2.

**Table 5:** Structural parameter estimates.

Hypothesized path	Original sample (O)	Sample mean (M)	Standard deviation (STDEV)	T statistics	*f*^2^	Confidence intervals	
						2.5%	97.5%
Direct effect							
SAT - > Motv	0.123	0.123	0.048	2.539*	0.02	0.347	0.556
SAT - > PsyCap	0.339	0.339	0.045	7.478***	0.130	0.029	0.216
PsyCap - > Motv	0.467	0.467	0.046	10.104***	0.265	0.250	0.426
Indirect effect							
SAT - > PsyCap - > Motv	0.158	0.159	0.028	5.650***		0.107	0.216
Total effect							
SAT - > Motv	0.281	0.282	0.050	5.571***		0.183	0.377

*f*^2^, effect size, SAT, satisfaction with academic majors; PsyCap, psychological capital; Motv, achievement motivation; ***: *p* < 0.001, *: *p* < 0.05.

As shown in Figure 2 and Table 5, the analysis of direct, indirect, and total effect relationships between the study’s constructs revealed that all estimated paths were positive and significant, resulting in the acceptance of all hypotheses. Hypothesis H1, which suggested that satisfaction with academic majors is positively associated with students’ achievement motivation (direct effect), was supported (*β* = 0.123, *t*-value = 2.539, *p* < 0.05). Hypothesis H2, suggesting a positive association between SAT and PsyCap, was also supported by the findings (*β* = 0.339, *t*-value = 7.478, *p* < 0.001). Further, The PLS-SEM findings confirmed H3, indicating that PsyCap is significantly associated with students’ achievement motivation (*β* = 0.467, *t*-value = 10.104, *p* < 0.001).

A bootstrapping procedure was conducted to examine the potential intermediating effect of PsycCap on the link between SAT and students’ motivation for achievement (indirect effect). Table 5 underscores that satisfaction with academic majors is significantly positively associated with students’ achievement motivation through PsyCap (*β* = 0.158, *t*-value = 5.650, *p* < 0.001). Hence, H4, which predicted a significant intermediating role of PsyCap in the nexus between SAT and achievement motivation, is accepted. When considering the total effect—both direct and indirect—of SAT on achievement motivation, the total path coefficient was *β* = 0.281 (*t*-value = 5.571, *p* < 0.001), showing a substantial association between SAT and achievement motivation through both direct and mediated pathways.

According to Zhao et al. (2010), full mediation occurs when only the indirect effect is significant, with an insignificant direct effect. In contrast, partial mediation is supported when both direct and indirect effects are significant. Since both the direct and indirect paths between SAT and achievement motivation were significant, PsyCap partially mediates this relationship, suggesting that while SAT enhances achievement motivation, a substantial portion of this effect occurs through PsyCap, reinforcing the importance of PsyCap as a substantial mediator in the link between SAT and achievement motivation.

The results of the study also demonstrated the effect size (*f^2^*) in accordance with Cohen (1988) guidelines indicating that the *f^2^* value for the effect of SAT on achievement motivation was 0.02, indicating a small effect size. This suggests that while satisfaction with academic majors has a significant relationship with achievement motivation, its direct impact is relatively modest. Further, the *f^2^* value of the SAT on PsyCap was 0.13, which falls close to the medium effect size threshold (0.15). This result highlights that satisfaction with academic majors plays a more substantial role in shaping students’ PsyCap, suggesting that students’ perceptions of their academic major may significantly influence their psychological resources, such as optimism, resilience, and self-efficacy. In addition, the *f^2^* value for PsyCap on achievement motivation was 0.265, representing a medium to large effect size. This finding indicates that PsyCap exerts a considerable impact on achievement motivation, demonstrating its critical role in enhancing students’ motivation to achieve academic goals.

## 5 Discussion

In this study, we seek to empirically investigate the link between students’ satisfaction with academic majors and achievement motivation among tourism and hospitality students, with a focus on the critical role of positive psychological constructs. Further, to explore the intermediating role of PsyCap in this link. Using PLS-SEM with the bootstrapping technique, the direct and indirect links between these constructs were assessed. The findings of this analysis suggested that the SAT is significantly associated with achievement motivation among tourism and hospitality students. This finding offers insights into factors that drive student achievement motivation in this field. This result aligns with existing literature (i.e., Subandi and Hamid, 2021; Suswanto et al., 2017; Ritonga et al., 2023; Baek et al., 2007) which indicate that satisfaction with their academic majors makes students more inclined to be motivated in pursuing their educational and career objectives. While the strength of the relationship (*β* = 0.123) is modest, it highlights that SAT could be an important, though not the sole, factor influencing achievement motivation. Drawing on existing literature, several additional factors are identified that may play a more significant role in student achievement motivation. These include environment-based education; sociocultural factors, collaborative team teaching, and home environment (social support from family) (Athman and Monroe, 2004; Wang et al., 2020; Anwar et al., 2021; Muola, 2010). While these studies are regionally focused, our findings from Saudi Arabia contribute to the literature by offering insights specific to the Middle Eastern context, particularly within tourism and hospitality higher education.

Further, academic major satisfaction is significantly associated with students’ PsyCap. This significant positive relationship underscores SAT’s potential role in fostering a positive psychological state among students in tourism and hospitality programs including self-efficacy, hope, resilience, and optimism. This finding align with previous contributions (i.e., Chi and Lee, 2022; Elom et al., 2023) which suggest that SAT may serve as a key factor linked to students’ PsyCap. Based on this finding, it could be concluded that students who report higher satisfaction with their academic major tend to exhibit greater confidence in their ability to succeed in their studies (self-efficacy), experience higher levels of hope, characterized by their ability to set clear goals and identify pathways to achieve them (hope), enhanced ability to bounce back from setbacks and maintain their motivation (resilience), and remain optimistic, even when facing challenges (optimism). This connection likely arises from the alignment between students’ interests and academic responsibilities, which could boost their confidence in navigating academic challenges and achieving their goals. Such satisfaction might also equip students to better manage the evolving demands of their fields, fostering a stronger commitment to their chosen discipline and a determination to overcome obstacles. It also could encourage a proactive mindset, enabling students to view potential setbacks as personal and academic growth opportunities.

Additionally, PsyCap was significantly associated with higher levels of students’ achievement motivation. This finding provides valuable insight into the psychological factors that may drive student motivation in educational settings, particularly tourism and hospitality programs. This significant positive relationship highlights the potential role of PsyCap, which comprises self-efficacy, hope, resilience, and optimism, in supporting students’ motivation to achieve their academic and career goals. This finding is in agreement with the previous findings which concluded that students with higher levels of PsyCap, tend to be more motivated to pursue their academic goals (Wenjiao and Wanbing, 2021; Datu et al., 2018; Adil et al., 2020; Saman and Wirawan, 2021; Herdem, 2019).

This finding suggests confident students are more likely to engage actively with their coursework, seek help when needed, and persist through challenges (Meng and Zhang, 2023; Abdolrezapour et al., 2023). In tourism and hospitality higher education, where practical experience and academic knowledge are crucial, self-efficacy appears to support students in addressing theoretical and hands-on challenges, potentially leading to greater academic success. Moreover, students with high levels of hope seem more inclined to adopt a proactive approach to their studies, including seeking internships, networking, and skill-building opportunities aligned with their career goals (Rand et al., 2020; Marques et al., 2017). Additionally, resilient students are more likely to perceive failures or challenges as temporary rather than impossible, fostering a determination to address obstacles and maintain their motivation to pursue academic and career aspirations (Abdolrezapour et al., 2023). Finally, optimistic students are likely to approach their studies with enthusiasm and maintain confidence in their potential for success (Hoy et al., 2006; Riveiro, 2014).

Finally, the study’s findings suggest that PsyCap plays a partially significant intermediating role in the nexus between students’ SAT and their motivation for achievement. This intermediate role highlights the positive psychological mechanisms through which SAT contributes to achievement motivation, suggesting that tourism and hospitality students who are satisfied with their academic majors could experience higher levels of PsyCap, which might subsequently enhance their motivation to achieve their academic and career goals. Moreover, the partial mediation of PsyCap revealed that while PsyCap does serve as an essential pathway linking SAT to achievement motivation, it is possible that other psychological, environmental, or situational factors could also contribute to motivating students. For instance, external sources of motivation, such as sociocultural factors, including cultural and gender differences, may independently contribute to students’ drive, regardless of their PsyCap levels (Wang et al., 2020). Additionally, academic and social motivational factors such as teachers’ instructional practices and social support from peers, faculty, or family could directly affect achievement motivation, acting as motivators outside the influence of PsyCap (Wentzel and Wigfield, 1998; Muola, 2010).

In summary, this study contributes to the broader literature on positive higher education by highlighting how satisfaction with academic majors and PsyCap interact to foster students’ achievement motivation. These findings underscore the importance of designing educational environments that promote student hope, self-efficacy, optimism, resilience, and intrinsic motivation, ultimately empowering them to achieve academic and career success.

## 6 Theoretical and practical implications

### 6.1 Theoretical implications

The study’s findings provide empirical support for integrating positive psychological resources (i.e., PsyCap) in understanding student motivation in educational settings, particularly tourism and hospitality higher education. The significant relationships between study constructs are in alignment with the positive psychology framework emphasis on fulfilling core psychological needs to foster intrinsic motivation. By enhancing students’ satisfaction with their academic major, educators can promote higher levels of PsyCap, including self-efficacy, hope, resilience, and optimism, which are crucial for achievement motivation. Further, the study contributes to the existing literature by highlighting the role of academic major satisfaction as a critical determinant of PsyCap. It suggests that the SAT is not just a matter of preference or contentment but also a crucial factor that enhances psychological resources. This expands the conceptual understanding of academic major satisfaction beyond mere contentment, positioning it as a fundamental factor in developing positive psychological states among students.

Moreover, the partially significant mediating role of PsyCap between academic major satisfaction and achievement motivation offers a nuanced perspective on how psychological factors influence student motivation. This suggests that while academic satisfaction directly associated with achievement motivation, PsyCap is an important pathway that enhances this effect. Lastly, this study proposes and empirically examines a model linking major satisfaction, PsyCap, and achievement motivation, contributing to the psychological and educational literature in tourism and hospitality higher education. The findings offer valuable insights, as the model provides a basis for future research aimed at exploring ways to promote PsyCap and achievement motivation in educational settings specific to tourism and hospitality.

### 6.2 Practical implications

The study’s findings suggest several key considerations for educators and program designers in tourism and hospitality higher education. Prioritizing academic major satisfaction is essential for educational institutions that seek to develop well-rounded, psychologically resilient graduates. This goal can be supported by designing curricula that reflect current industry needs and trends, incorporating hands-on courses, certifications, and practical skills. Such elements equip students with real-world readiness and contribute to greater satisfaction with their program (Marinakou and Giousmpasoglou, 2013). Further, integrating real-world experiences, such as internships, industry partnerships, and field visits, allows students to apply theoretical knowledge practically, deepening their understanding and commitment to the field (Hussien and Lopa, 2018). Creating inclusive, interactive, and supportive learning environments through active learning methods, personalized feedback, and tailored academic advising to help students set, track, and achieve academic and career goals is critical for student satisfaction. Moreover, establishing regular channels for student feedback on courses, teaching methods, and program content is essential for enhancing satisfaction and fostering positive PsyCap (Kim and Jeong, 2018; Chau and Cheung, 2018; Huang et al., 2023).

Given the significant role of PsyCap in mediating the relationship between satisfaction with academic major (SAT) and achievement motivation, educational institutions should consider implementing targeted interventions to foster PsyCap development. For example, research (i.e., Li et al., 2023; Huang et al., 2023; Margaça et al., 2021) suggests that conducting workshops focused on core tourism and hospitality skills, such as customer service, problem-solving, and crisis management, can significantly boost students’ confidence in their abilities, leading to stronger self-efficacy (Nambudiri et al., 2020). Additionally, providing academic counseling services to help students manage workloads, prioritize tasks, and create balanced schedules supports the development of critical skills to overcome stressors and maintain motivation. Workshops in positive psychology can help students understand the benefits of a positive outlook, showing how optimism impacts motivation and perseverance in academic and career contexts. Furthermore, programs that teach practical techniques, including mindfulness, stress management, and adaptive coping skills, enable students to manage stress effectively, enhancing their capacity to handle academic challenges and setbacks (Saman and Wirawan, 2021; Herdem, 2019). Finally, pairing students with mentors who offer guidance on realistic career and academic goals provides valuable support, as mentors can help students navigate their paths, instilling a sense of hope and helping them envision a clear route to achieving their ambitions (Nambudiri et al., 2020).

## 7 Limitations of the study and further research

The study has several limitations: first, the sample was limited to undergraduate students from one governmental university in Saudi Arabia, specifically those in tourism and hospitality management, which might constrain the broader applicability of the results. Future research could compare different cultural contexts to enhance generalizability. Second, the cross-sectional design only captures data at one point in time, preventing the examination of changes over time and causal relationships. Longitudinal studies are recommended for a deeper understanding of these dynamics. Third, the study focused on specific psychological constructs (self-efficacy, hope, resilience, and optimism) within PsyCap, without considering other relevant factors like stress or anxiety. A broader scope could provide a more comprehensive understanding. Fourth, while the study examined both direct and indirect effects of academic major satisfaction on achievement motivation through PsyCap, it did not explore potential moderating factors that could influence these relationships. Future research could explore additional factors like gender, academic level, and other demographics to better understand how these characteristics influence students’ satisfaction with their major (SAT), psychological capital (PsyCap), and achievement motivation. Fifth, the exciting review revealed the reverse causality between PsyCap and satisfaction with academic majors. Accordingly, further research could explore alternative models to validate the robustness of the proposed theoretical framework. For instance, exploring the impact of PsyCap as a predictor of satisfaction with academic majors (the mediator) and achievement motivation (dependent variable) provides a different perspective on the interplay among these constructs.

## Data Availability

The original contributions presented in the study are included in the article/supplementary material, further inquiries can be directed to the corresponding author.
